# Glutathione – Hydroxyl Radical Interaction: A Theoretical Study on Radical Recognition Process

**DOI:** 10.1371/journal.pone.0073652

**Published:** 2013-09-09

**Authors:** Béla Fiser, Balázs Jójárt, Imre G. Csizmadia, Béla Viskolcz

**Affiliations:** 1 Department of Chemical Informatics, Faculty of Education, University of Szeged, Szeged, Hungary; 2 Department of Chemistry, University of Toronto, Toronto, Ontario, Canada; Instituto de Tecnologica Química e Biológica, UNL, Portugal

## Abstract

Non-reactive, comparative (2×1.2 μs) molecular dynamics simulations were carried out to characterize the interactions between glutathione (GSH, host molecule) and hydroxyl radical (OH^•^, guest molecule). From this analysis, two distinct steps were identified in the recognition process of hydroxyl radical by glutathione: catching and steering, based on the interactions between the host-guest molecules. Over 78% of all interactions are related to the catching mechanism *via* complex formation between anionic carboxyl groups and the OH radical, hence both terminal residues of GSH serve as recognition sites. The glycine residue has an additional role in the recognition of OH radical, namely the steering. The flexibility of the Gly residue enables the formation of further interactions of other parts of glutathione (e.g. thiol, α- and β-carbons) with the lone electron pair of the hydroxyl radical. Moreover, quantum chemical calculations were carried out on selected GSH/OH^•^ complexes and on appropriate GSH conformers to describe the energy profile of the recognition process. The relative enthalpy and the free energy changes of the radical recognition of the strongest complexes varied from −42.4 to −27.8 kJ/mol and from −21.3 to 9.8 kJ/mol, respectively. These complexes, containing two or more intermolecular interactions, would be the starting configurations for the hydrogen atom migration to quench the hydroxyl radical *via* different reaction channels.

## Introduction

**Figure 1:**
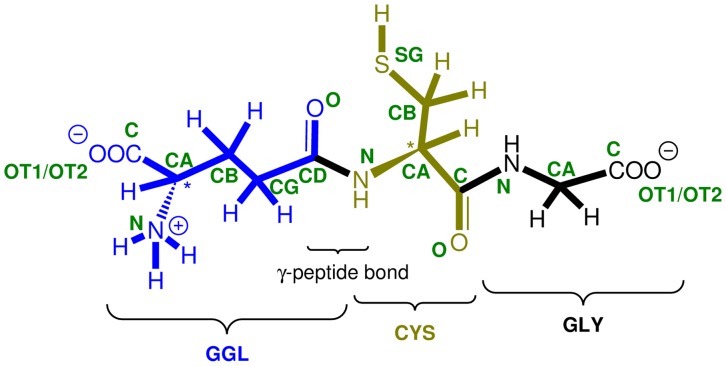
The chemical structure of GSH, with the atomic naming scheme (indicated with green).

Molecular recognition – the interaction between a larger host and smaller guest molecules – is one of the most important biochemical processes [1]. This complex mechanism can take place during intra- and intercellular communication, the induction of the immune system and the response to external stimuli *etc*. [2]. The cell itself evolves its own defensive mechanism against external actions, which can damage cells or cellular organelles, and this mechanism has been investigated for a long time. Free radical initiated oxidation is one of these external actions and one of the most important antioxidants [3], used as a defense mechanism, is a small tripeptide, glutathione (γ-L-glutamyl-L-cysteinyl-glycine, GSH, **Figure 1**).

GSH is a nonprotein free thiol present in high concentrations in the living organisms [4] and it is essential in a number of biochemical processes [5]–[7]. GSH exhibits antioxidant, radical scavenging activity by its electron donating ability [8], [9]. This enables GSH to neutralize free radicals, especially reactive oxygen species (ROS) such as the superoxide, hydroperoxyl, hydroxyl (OH**^•^**) radicals, having electron acceptor ability. The hydroxyl radical is one of the most reactive of the ROS [10] and has a key role in the oxidative stress related events, such as lipid peroxidation [11] and DNA oxidation [12], [13]. The rate constants of the reactions between GSH and radicals, as well as the GSH and hydroxyl radical reaction were studied previously by experimental methods, e.g. by laser photolysis, absorption spectroscopy and pulse radiolysis [9], [14]. The theoretical calculations were focused on the calculation of the potential energy surface [3] and the rate constants of elementary steps [15]. Furthermore, conformational analyses of GSH were carried out by NMR spectroscopy [16]–[18] and molecular dynamics (MD) [18]–[21] methods, which showed that GSH is flexible and does not adopt a strongly preferred conformation. Recently Machuqueiro *et al*. performed constant-pH MD simulations for GSH and GSSG (oxidized form of GSH) [20]. They concluded that the conformational flexibility of GSH is pH-dependent and it has reduced flexibility at higher pH (pH>10) values.

The large flexibility of GSH can be the weak point of the classical potential energy surface calculations, because the most reliable initial conformations are difficult to find. Moreover, the radical scavenging mechanism depends on the interactions formed between GSH and radicals, and the steric properties of collisions and attractive interactions can strongly influence the overall kinetics and the mechanism of this bimolecular reaction. To overcome this limitation, structures for further *ab initio* calculations could be determined by non-reactive molecular dynamics simulations. Therefore, we set a long lasting, comparative MD simulation for GSH and GSH/OH**^•^** systems. The MD trajectories will be able to characterize the different interactions between GSH and OH**^•^**. Moreover, the non-reactive MD trajectories combined with *ab initio* calculations allow us to describe a detailed free radical recognition process.

## Materials and Methods

The glutathione anion is found to be dominant at physiological pH, where the γ-L-glutamic acid predominantly exists in its zwitterionic form, while the carboxyl group of the glycine residue prefers to be deprotonated [21]. For these reasons, to obtain the most reliable theoretical model for GSH in water, its anionic form was considered and is hereinafter referred to as GSH.

Five independent molecular dynamics (MD) simulations (5×240 ns) were performed for the GSH and GSH/OH**^•^** systems, respectively. GSH was solvated with TIP3P [22] water molecules and one Na^+^ ion was also placed in the box in order to ensure the electro-neutrality of the system. The simulation box was cubic (37^3^ Å^3^), where the minimum distance between any atom of the GSH and the wall of the box was 12 Å. The simulations were conducted with the Desmond v. 30110 [23] software using the CHARMM22 [24] force field. The short range van der Waals and electrostatic cut-off values were set to 9.0 Å and the long-range electrostatic interaction was calculated *via* the Particle Mesh Ewald [25] method.

The missing bond parameters and charges for the OH**^•^** radical were calculated with the Force Field Toolkit Plugin [26] implemented in Visual Molecular Dynamics (VMD) [27].

The simulation protocol was as follows: 1) steepest descent minimization (with and without solute restraints); 2) NVT MD (T = 10 K, Δt = 12 ps) with the Berendsen thermostat [28] τ_T_ = 0.1 ps and restrained solute heavy atoms; 3) NPT MD (T = 10 K, Δt = 12 ps, p = 1 bar) with Berendsen thermo- and barostat (τ_T_ = 0.1 ps, τ_p_ = 50 ps, separate coupling for solute and solvent) and no restraints; 4) NPT MD (T = 310 K, Δt = 12 ps, p = 1 bar) with Berendsen thermo- and barostat (τ_T_ = 0.1 ps, τ_p_ = 50 ps, separate coupling for solute and solvent) and restrained solute heavy atoms; 5) NPT MD (T = 310 K, Δt = 24 ps, p = 1 bar) with Berendsen thermo- and barostat (τ_T_ = 0.1 ps, τ_p_ = 2.0 ps, separate coupling for solute and solvent) and no restraints. 6) NPT MD (T = 310 K, p = 1 bar) with Berendsen thermo- and barostat [28] (τ_T_ = 0.1 ps, τ_p_ = 2.0 ps, separate coupling for solute and solvent) and no restraints.

The structures were saved every 9.8 ps, which resulted in 25 000 frames for each simulation. The protocol was repeated 5 times with different random velocities and a total of 1.2 μs of simulations were obtained for each GSH and GSH/OH**^•^** system, respectively.

The metadynamics [29] method was used to reconstruct the free energy surface of interaction between GSH and OH radical. A 24 ns biased molecular dynamics simulation was performed with two collective variables: the distance between the oxygen of OH**^•^** and Gly.OT1 atom (d (OT1 – OH**^•^**) and distance between the oxygen of OH**^•^** and Cys.SG atom (d (SG – OH**^•^**). During the calculation, the height of the Gaussian was set to 0.03 kcal/mol, the width was set to 0.05 Å, and barriers were applied at 6 Å in order to prevent large changes in the direction of the variables.

Intra- and intermolecular interactions were identified by the geometric analysis using the following criteria: d(A×××D) <3.5 Å and α(A×××H-D) >100.0°. The structural analysis was performed with the *ptraj* module of the AmberTools 1.5 program package and Visual Molecular Dynamics [27] was used to prepare the 3D structures in the figures.

To describe these interactions more in detail, electron density analyses (Atoms in Molecules, AIM) [30], [31] were carried out on some geometrically selected structures at the B3LYP/6-31G (d) level of theory. The AIM analysis was carried out with the AIM2000 program [32]. Previously, similar refinements were successfully used to improve the analysis of intramolecular interactions in the case of human galactokinase enzyme [33].

Geometry optimization were conducted on properly selected GSH/OH**^•^** complexes and GSH conformers with the BHandHLYP density functional combined with the 6–31G(d) split valence basis set. To mimic the bulk water, the solvation model “D” (SMD) [34] was used. Normal mode analysis was also carried out at the same level of theory in order to confirm that the structures obtained are minima on the respective potential energy surface. The quantum chemical calculations were carried out using the Gaussian 09 program package [35].

## Results and Discussion

Interactions between water and radicals have been investigated earlier by classical MD methods [36], wherein the authors used the same van der Waals parameters for the radicals (OH**^•^**, HO_2_ **^•^**) as for the water molecules. The obtained free energy profiles through the water slab were in good agreement with experimental studies; therefore we developed new parameters only for the O-H bond term and the O and H partial charges. Atomic point charges of the OH radical were calculated to be 0.444 for H and −0.444 for O and the bond parameters for the O-H bond were found b_0_ = 0.9791 Å and K_b_ = 444.8474 kcal/molÅ^2^. Other consequences of the above mentioned paper [36] are that a direct reaction between water molecules and radicals is not necessary in order to describe the interaction between them. Therefore, to investigate the interaction patterns between GSH and OH**^•^**, we can use classical MD simulations to generate different GSH conformers and identify those parts of the molecule that are the most important molecular recognition sites.

**Figure 2:**
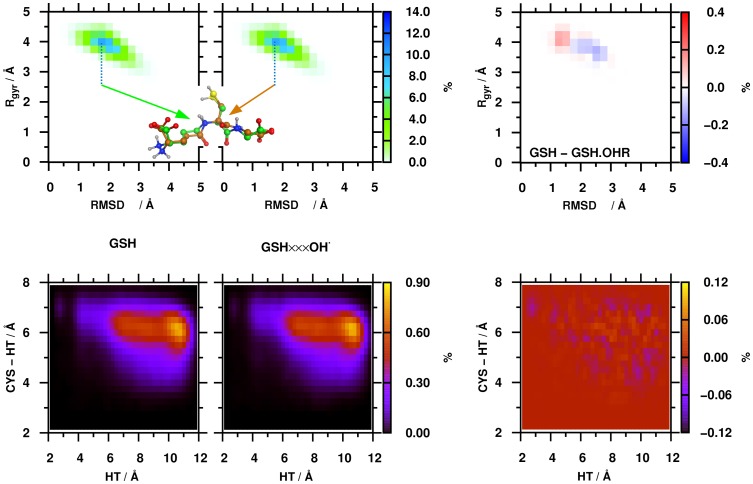
The percentage distribution of the structures on the RMSD – R_gyr_ (upper panel) and HT – CYS-HT surfaces was calculated for the GSH and the GSH/OH^•^ systems (left panels). Representative structures from the most populated region from the RMSD – R_gyr_ surface are shown as well. The different origin of the representative structures is indicated by colored carbon atoms (green – GSH, brown – GSH/OH^•^). The differences between surfaces were also calculated (right panel).

The root-mean-square deviation (RMSD) of the heavy atoms was calculated for the glutathione structures obtained with respect to the initial, extended structure of GSH (see **Figure S1**). The RMSD value fluctuated between 1.00 and 3.00 Å, indicating that the GSH was indeed very flexible during the simulations, which is in good agreement with previous studies [18]–[21]. The compactness of the GSH structure during the simulations was measured by the radius of gyration of heavy atoms (R_gyr_). The R_gyr_ values for most of the GSH structures varied from 3.0 to 4.5 Å both with and without hydroxyl radical. The percentage of the distribution of the structures on the RMSD – R_gyr_ surface was also calculated (**Figure 2**). In order to describe the flexibility of GSH from another structural point of view we used the same parameters as Machuqueiro et al. [20]. All possible distances were measured between the Gly.OT1/OT2 and GGL.OT1/OT2/N and the minimum value among these 6 distances was used as the head-tail distance (HT). The other parameter was the minimum distance between CYS.SG and the above mentioned atoms (CYS-HT). Thereafter, using a 0.25 Å bin width, the distribution of the structures on this surface was determined.

Similar distributions were obtained for the simulations with and without OH**^•^**, which can be seen on the difference surfaces (right panel, **Figure 2**). The largest deviation obtained was only about ±0.4% and ±0.1% on the RMSD – R_gyr_ and HT – CYS-HT surfaces, respectively. In case of the HT – CYS-HT surface, the same distribution was obtained also in our case comparing the results of Machuqueiro *et al*.: the HT values varied between 2 and 12 Å, while the CYS-HT values were between 2 and 8 Å.

This indicates that the presence of OH**^•^** has no large impact on the dynamic nature of GSH in a non-reactive, classical MD simulation.

The most populated regions of the GSH and GSH/OH**^•^** systems (12.9% and 13.1% of the structures, respectively) are located in the same [1.75 Å ≤ RMSD <2.00 Å; 3.75 Å ≤ R_gyr_ <4.00 Å] range of the RMSD – R_gyr_ surface. The averages of the RMSD and the R_gyr_ values in this most populated range were calculated and the structures which had the smallest deviation from the averages were selected for comparison as representative structures. The RMSD between the two representative GSH structures is 1.34 Å, which means that the representative structures fit to each other very well (**Figure 2**). The largest deviation was obtained for the glycine (GLY) residue and the peptide bond between the cysteine (CYS) and GLY residues. In the representative structures we did not obtain any intramolecular hydrogen bonds, except the interaction between the protonated amine and the deprotonated caboxyl group in the γ-glutamic acid (GGL) residue. Furthermore, the representative structures of GSH are in good agreement with the conformations obtained previously by NMR and MD studies [17]–[21].

Intramolecular hydrogen bond analyses were carried out as well for GSH in both systems for each snapshot from the MD simulations. The intramolecular hydrogen bonds were formed in less than 0.05% of the total number of the MD frames. This also indicates that the structure of glutathione is very flexible, since internal interactions cannot facilitate a preferred conformation. These results are in good agreement with other studies, where the authors concluded that in tripeptides the intramolecular H-bonding interactions do persist *in vacuo* or in acetonitrile, but vanish in water [37], [38]. These findings suggested that in aqueous phase the solute – solvent interactions are favored instead of intramolecular H-bonds, therefore in the following sections we focused on the analysis of GSH×××OH**^•^**/WAT interactions (××× – denotes non-covalent interactions).

**Figure 3:**
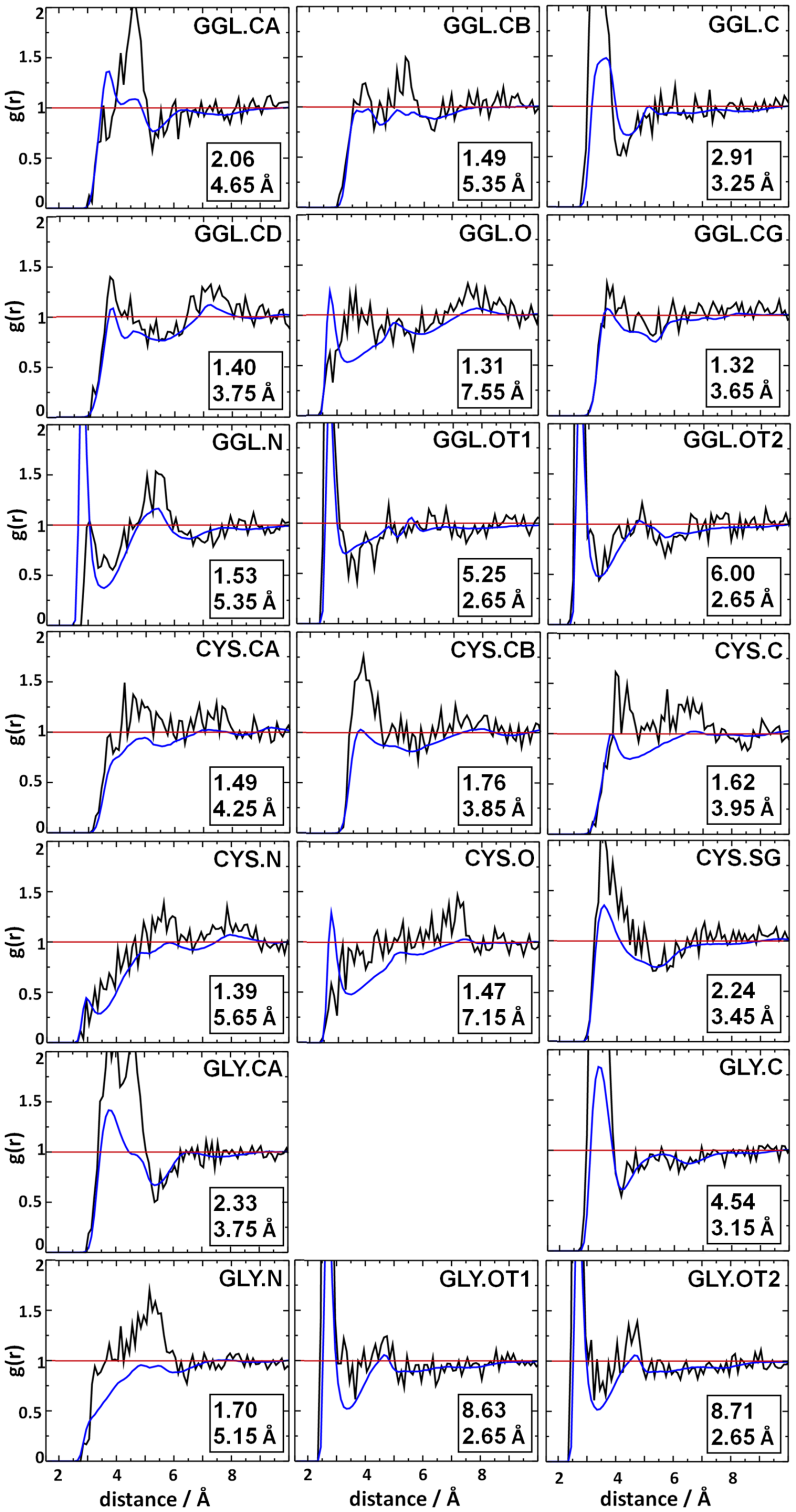
The radial distribution functions (*g(r)*) were calculated between the oxygen atom of water (blue curves) or OH^•^ (black curves) and all heavy atoms in GSH for the GSH/OH^•^ system. The maximum *g(r)* values and the corresponding distance for OH^•^ are indicated in the lower right corner of each graph.

During the MD simulation, the individual atomic pair distances and distributions can be calculated by the radial distribution function (*g(r)*) between the oxygen atom of water or OH**^•^** and all heavy atoms in GSH (**Figure 3**).

We considered those heavy atoms as the most probable radical attack points of GSH where the *g(r)* values show high maxima for OH**^•^**. During the *g(r)* curve analysis and in the further discussion all CA atoms were considered, although in the case of Gly the terminal carboxyl group is responsible for the enrichment around this region. Nevertheless, we were interested in these regions as well, because the α-carbon atoms are highly vulnerable attack points in the proteins [39]. Hydrogen atom abstraction by radicals from these positions can easily happen if the α-carbons are accessible, because the process is favorable from the thermodynamics point of view [40]. In the case of the γ-glutamic acid residue, the OH**^•^** enrichment is conspicuous near the α-carbon (GGL.CA), the carboxyl carbon (GGL.C) and both carboxyl oxygens (GGL.OT1/GGL.OT2). The β-carbon (CYS.CB) and the sulfur (CYS.SG) in the cysteine residue are potential radical attractors, as shown by the corresponding *g(r)* values. The α-carbon (GLY.CA), the carboxyl carbon (GLY.C) and carboxyl oxygens (GLY.OT1 and GLY.OT2) in the glycine residue also have radical control ability. The distribution functions of the GLY.OT1/2 show the same characteristics as the corresponding *g(r)*s of the GGL residue (see **Figure** **3**). To determine the probability of finding the OH**^•^** molecule near GSH, the maxima of the *g(r)* curves (*g(r)*max) were analyzed and these values were collected in **Table 1** with the corresponding distances.

**Table 1:** The maximum values of the radial distribution functions (*g(r)*) between the oxygen atom of OH^•^ and all heavy atoms in GSH.

Res.	Atom	r (Å)	*g(r)*max	Norm.
GGL	CA	4.65	2.06	1.57
	CB	5.35	1.49	1.14
	C	3.25	2.91	2.22
	CD	3.75	1.40	1.06
	O	7.55	1.31	1.00
	CG	3.65	1.32	1.01
	N	5.35	1.53	1.17
	OT1	2.65	5.25	4.01
	OT2	2.65	6.00	4.58
CYS	CA	4.25	1.49	1.14
	CB	3.85	1.76	1.34
	C	3.95	1.62	1.23
	N	5.65	1.39	1.06
	O	7.15	1.47	1.12
	SG	3.45	2.24	1.71
GLY	CA	3.75	2.33	1.78
	C	3.15	4.54	3.47
	N	5.15	1.70	1.30
	OT1	2.65	8.63	6.58
	OT2	2.65	8.71	6.65

The normalized values (Norm.) were calculated (Norm.  = *g(r)*max/min[*g(r)*max]) and are also tabulated.

Based on these *g(r)*max values, the most probable OH**^•^** attack points can be selected. The highest *g(r)*max values for the OH**^•^** belong to the carboxyl oxygens of GLY (8.63 and 8.71). These were followed by the carboxyl oxygens of GGL (5.25 and 6.00), while the lowest value (1.31) was attributed to the carbonyl oxygen of GGL (GGL.O). Compared to the probability at the carbonyl oxygen, we obtained more than 1.5 times higher probability values around the α-carbons (GGL.CA and GLY.CA), the carboxyl carbons, oxygens (GGL.C, GLY.C and GGL.OT1,2, GLY.OT1,2) and the sulfur (CYS.SG), altogether around 9 atomic positions. Seven of these cases are situated closer than the maximum distance criteria of the hydrogen bonding interaction (<3.5 Å). The high probability of finding of OH**^•^** around the carboxyl carbons (GLY.C and GGL.C) is caused by the strong H-bonds between OH**^•^** and negatively charged carboxyl oxygen atoms. Therefore we will omit these from the further analysis, since there are no direct interactions between OH**^•^** and these carbon atoms.

To obtain a more detailed description about the interactions between the hydroxyl radical and the different functional groups of GSH, interaction pattern analyses were carried out. The interactions were determined between the OH**^•^** and those heavy atoms of GSH where the *g(r)* curves showed higher probability of finding it, and/or which were energetically favorable attack points (GGL: CA, N, OT1, OT2; CYS: SG, CA, CB and GLY: CA, OT1, OT2). All in all, 2305 structures were found where at least one interaction was established between the OH**^•^** radical and the corresponding parts of GSH. The most frequently occurring interaction (∼78%) was present between the OH**^•^** and the anionic carboxyl groups of GSH. Due to the fact that the -SH group is responsible for the antioxidant activity of GSH, we determined the structures in which the OH**^•^** is interacting with the sulfhydryl group (∼4%) as well.

**Figure 4:**
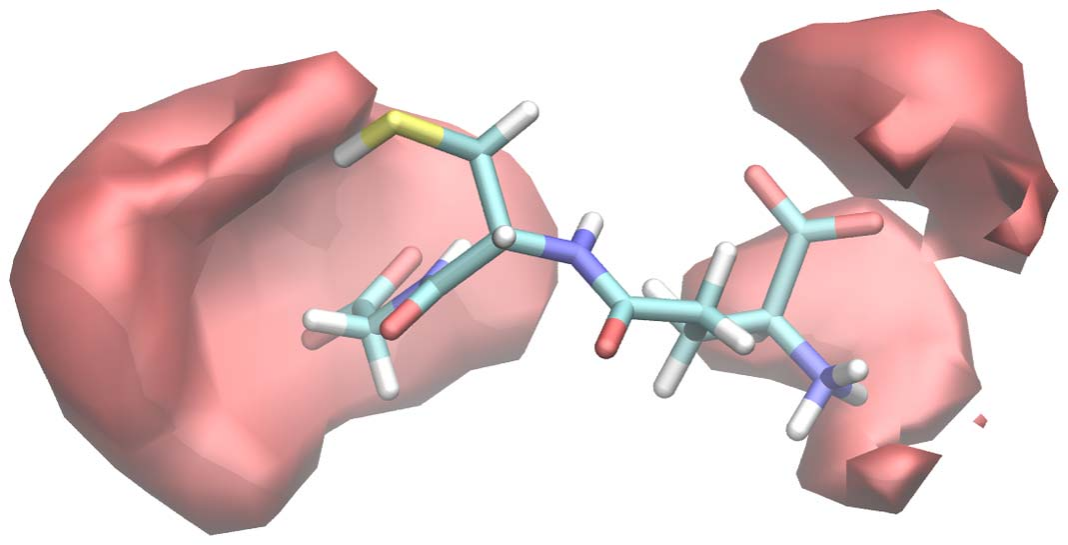
The volumetric map was created for the radical (OH^•^) occurrence around those 838 structures where the OH^•^ interacts with at least two heavy atoms of the GSH.

In around 36% (838) of the structures, the OH**^•^** interacts with at least two heavy atoms of GSH. The distribution of OH**^•^** around GSH in such structures was depicted as a volumetric map (see **Figure 4**).

The volumetric map also demonstrates that the carboxyl oxygens (GGL.OT1,2 and GLY.OT1,2) of GSH are dominant in the interactions, and that the OH**^•^** is mostly hydrogen-bonded with these atoms of GSH. The thiol (CYS.SG) and the protonated amine group (GGL.N) are also important for intermolecular interactions between GSH and OH**^•^**. 82% of these structures contained hydrogen bonds where the OH**^•^** is interacting with carboxyl oxygens. These structures can be divided into two subgroups, depending on which site of GSH (GLY or GGL) takes part in the interaction (GLY ∼48%, GGL ∼34%). In 11% of the structures, the hydroxyl radical formed interactions with a carboxyl oxygen and with a hydrogen bonded atom at the same time.

**Figure 5:**
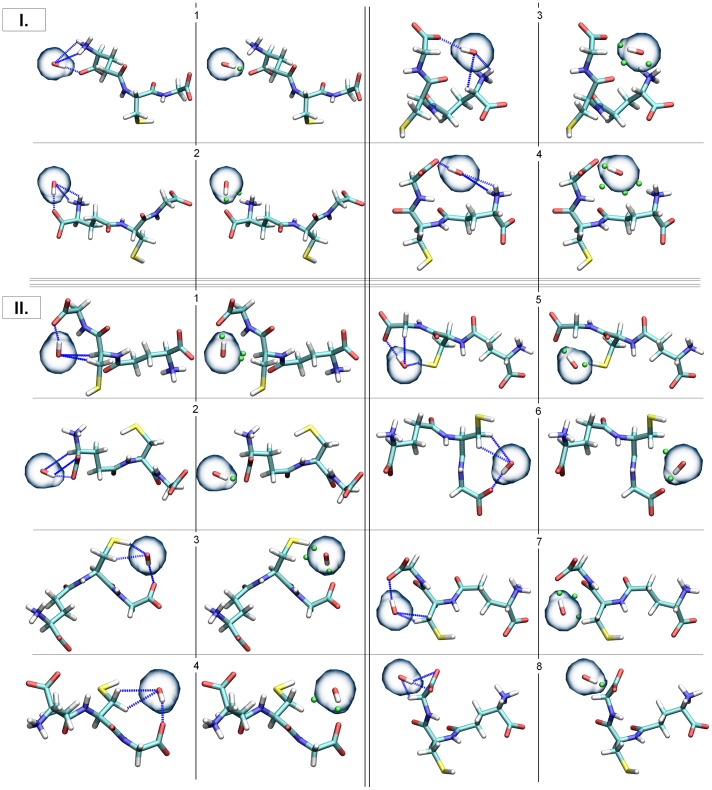
The structures that contained the maximum (3) number of interactions between GSH and the OH^•^ based on the geometrical criteria are depicted (I. – zwitterionic group, II. – anionic group). The interactions based on geometrical criteria are indicated with blue, dashed lines, while those resulted from the AIM analyses are depicted with green points (bond critical points, BCPs).

The analysis of the intermolecular interactions revealed 12 cases in which three interactions were established between GSH and OH**^•^** (see **Figure 5**), and this is the highest number of such (GSH×××OH**^•^**) intermolecular interactions. These are the most stable complexes, because of the high number of interactions between the two molecules. For this reason, AIM analysis was carried out on these structures to describe these interactions and investigate their existence from a molecular electron density point of view (see **Figure 5**, second and fourth columns).

Two main types of interactions can be identified in these cases, zwitterionic and anionic, based on the interacting functional groups of GSH (see **Figure 5** **,** I. and II.). The zwitterionic group (−NH_3_^+^×××OH**^•^**×××−CO_2_^−^) contains 4 members, where one of the anchor points is the protonated amine group of GGL (GGL.N) and the other one is a deprotonated carboxyl group. In the anionic group (−CO_2_^–^×××OH**^•^**×××−X-H) there are 8 structures where the main interaction between OH**^•^** and GSH is formed with the carboxyl group of the GLY or the GGL residue. Besides this interaction, others were also found in these complexes with the comprising of GGL.CA or GLY.CA and/or CYS.CA/CB/SG. The AIM analysis resulted in bond critical points (BCPs) indicating the existence and the strength of the interactions. The BCPs varied from 0.002 a.u. to 0.058 a.u. (higher electron densities at the BCPs represent stronger interactions). The weakest interactions correspond to geometrically not investigated ones (GLY[–N-HN]×××OH**^•^**), which appeared in two cases (I/4, zwitterionic group and II/7, anionic group). The AIM analysis showed that the strongest interactions (H-bonds) were between the carboxyl oxygens of the glycine and the hydroxyl radical (GLY.OT1,2×××OH**^•^**). This finding is in good agreement with the g*(r)*max values calculated.

**Figure 6:**
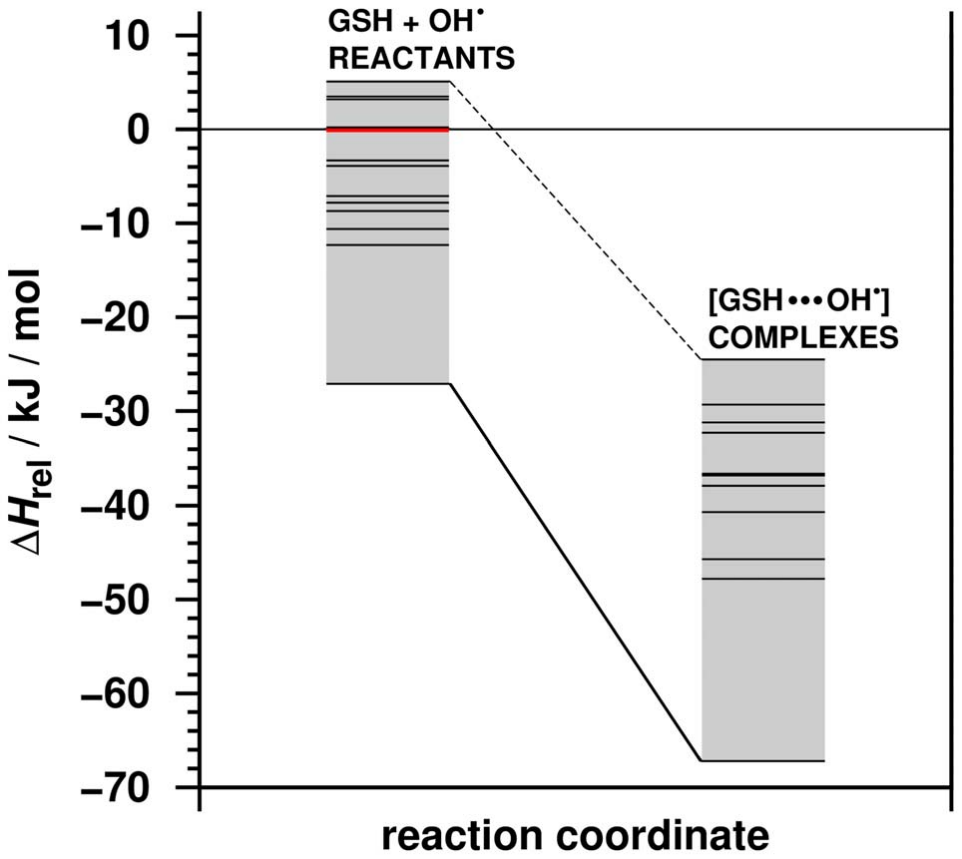
The relative enthalpy of the optimized GSH conformers and the GSH/OH^•^ complexes. The reference conformer (red line) was the representative structure obtained from the molecular dynamics simulation of GSH without OH**^•^**. The calculations were carried out at the BHandHLYP/6-31G(d) level of theory combined with the SMD implicit (continuum) solvent model.

Geometry optimization and frequency calculations were conducted on selected GSH/OH**^•^** complexes and GSH conformers at the BHandHLYP/6-31G(d) level of theory to describe the energy profile of the recognition process. The representative GSH structure from the molecular dynamics simulation of GSH without OH**^•^** was selected as a reference structure. The relative enthalpy of other conformers with respect to this structure is in the range of −27.1–5.1 kJ/mol (see **Figure 6**).

The lowest enthalpy conformer is the 3^rd^ from the zwitterionic group (see **Figure 5**, right upper corner). The complex formation between the hydroxyl radical and the GSH is highly exothermic, regardless of the molecular motion of GSH, as well as the attack point of OH**^•^** on the glutathione. The enthalpy of complex formation is between −42.4 and −27.8 kJ/mol. The relative enthalpy of the formed complexes varies from −67.2 to −24.5 kJ/mol compared to the reference conformer and the hydroxyl radical. These results show that the radical recognition process of GSH (GSH/OH**^•^** complex formation) is energetically favorable.

In summarizing our results, we outline a possible general radical recognition process as follows. The *g(r)* curves show that the probability of finding OH**^•^** is 4 times larger around the GLY.OT1,2 (*g(r)*max >8.6) compared to CYS.SG (*g(r)*max  = 2.2), which is the common antioxidant part of GSH. Additionally, the relative amount of the GLY.OT1,2×××OH**^•^** H-bonds compared to the total number of interactions shows us that the terminal GLY may have a double role in the radical recognition: catching and steering. In the first step of the process, the GLY.OT1,2 most often forms a H-bonded intermolecular complex with OH**^•^**, however the GGL.OT1,2 and/or GGL.N are also capable of this, quasi catching the radical from the bulky solvent phase. In the second step, owing to the high flexibility of GLY, it can further steer the radical in the direction of the CYS. In this step, new interactions are forming, e.g. with the CYS.CB, and the original interactions (GLY.OT1,2×××OH**^•^**) also remain. In our previous work [3] we showed that the bond dissociation energies of the hydrogen atoms from CB are one of the highest among all possibilities. Therefore, hydrogen abstraction from this β-carbon atom is unlikely. The OH**^•^** does not stop here, but continues to move forward on the way to the –SH group. After the radical recognition, the detoxification of OH**^•^** by GSH can take place, the OH**^•^** is forwarded to CYS.SG and abstracts a hydrogen from this group.

**Figure 7:**
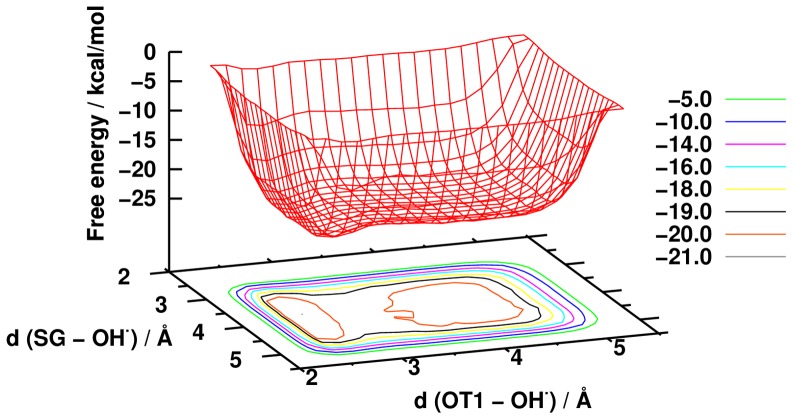
The free energy surface of the GSH×××OH^•^ radical interaction determined by metadynamics calculation.

The recognition mechanism is based on selected structures from a large ensemble, therefore one may assume that it lacks the statistical relevance. We have to emphasize here that during the calculations we saved the structures at every 9.8 ps to sample diverse conformations. In order to confirm further our results we have several possibilities. We can perform additional calculations with more frequent structure sampling, or we can focus around the region of catching and steering. We decided to use a biased MD simulation by means of metadynamics using two collective variables. Based on this calculation the free energy surface of the recognition mechanism was determined (**Figure 7**).

As one can see in **Figure 7**, if we use the above mentioned collective variables, we obtain a deep free energy valley with two minima. The first minimum is located on the surface between 3 Å < d (OT1 – OH**^•^**) <4.2 Å and 3.8 Å < d(SG – OH**^•^**) <4.8 Å and a local barrier at d(OT1 – OH**^•^**)  = 3.0 Å with a height lower than 1 kcal/mol. Data obtained from unbiased MD calculations indicate that the OH radical can easily access this local maximum because we obtained a sharp peak on the g(r) curve at 2.65 Å. The second minimum lies in the 2.4 Å < d(OT1 – OH**^•^**) <2.8 Å and 3.5 Å < d(SG – OH**^•^**) <4.8 Å range. This second minimum valley confirms our proposed steering mechanism: after catching the OH**^•^** by one of the carboxylate oxygen of Gly, and due to its high flexibility, GLY can steer OH**^•^** toward the thiol group of Cys. In this process we did not obtain any barrier, indicating that there is no thermodynamic control regarding steering. The lowest free energy point lies at 2.65 Å (d(OT1 – OH**^•^**)) 3.65 Å (d(SG – OH**^•^**)) on the surface and two of the selected structures correspond to this point: the 3^rd^ and the 5^th^ structure from the anionic group (see **Figure 5**).

## Conclusions

Two, in total 1.2 μs comparative MD simulations were conducted on GSH and GSH/OH**^•^** systems to explore the molecular recognition process and identifying the OH radical attractor regions of GSH.

The high flexibility of GSH was preserved during the simulation of the GSH/OH**^•^** system and this is one of the driving forces of the radical recognition process. Two main steps of the detailed molecular radical recognition process of GSH were assigned, namely catching and steering. In ∼78% of all interactions characterized, strong complexes were formed between anionic carboxyl groups and the OH radical. After the catching step, the strong carboxyl-OH^•^ complexes could evolve additional interactions with the other parts of GSH, stabilized by both the donor and acceptor features of the OH radical.

The glycine residue dominates the steering role in the recognition step, while the glycine-hydroxyl radical complexes could facilitate further interactions with the thiol group, α- and β-carbons of the cysteine residue *via* the OH**^•^** lone pair electron. The glutamic acid residue does not show this property during the MD simulations. Quantum chemical calculations on selected GSH/OH**^•^** complexes revealed exothermic heats of formation between −42.4 and −27.8 kJ/mol, and these strong complexes become the starting configurations of individual bond rearrangement to complete the radical scavenging mechanism.

## Supporting Information

Figure S1 The RMSD of the heavy atoms of the GSH along the 5×240 ns long trajectories in the case of GSH and the GSH/OH^•^ systems.
(TIF)
